# Africanization of a feral honey bee (*Apis mellifera*) population in South Texas: does a decade make a difference?

**DOI:** 10.1002/ece3.1974

**Published:** 2016-03-02

**Authors:** Juliana Rangel, Melissa Giresi, Maria Alice Pinto, Kristen A. Baum, William L. Rubink, Robert N. Coulson, John Spencer Johnston

**Affiliations:** ^1^ Department of EntomologyTexas A&M University2475 TAMUCollege StationTexas77843‐2475; ^2^ Department of BiologyTexas A&M University3258 TAMUCollege StationTexas77843‐3258; ^3^ Mountain Research Centre (CIMO)Polytechnic Institute of BragançaCampus de Sta. ApolóniaApartado 1172Bragança5301‐855Portugal; ^4^ Department of ZoologyOklahoma State University501 Life Sciences WestStillwaterOklahoma74078; ^5^ P.O. Box 2686EdinburgTexas78540; ^6^ Knowledge Engineering LaboratoryDepartment of EntomologyTexas A&M UniversityCollege StationTexas77843‐2475

**Keywords:** *Apis mellifera*, feral Africanized honey bee, hybridization, introgression, mitochondrial DNA, nuclear DNA

## Abstract

The arrival to the United States of the Africanized honey bee, a hybrid between European subspecies and the African subspecies *Apis mellifera scutellata*, is a remarkable model for the study of biological invasions. This immigration has created an opportunity to study the dynamics of secondary contact of honey bee subspecies from African and European lineages in a feral population in South Texas. An 11‐year survey of this population (1991–2001) showed that mitochondrial haplotype frequencies changed drastically over time from a resident population of eastern and western European maternal ancestry, to a population dominated by the African haplotype. A subsequent study of the nuclear genome showed that the Africanization process included bidirectional gene flow between European and Africanized honey bees, giving rise to a new panmictic mixture of *A. m. scutellata‐* and European‐derived genes. In this study, we examined gene flow patterns in the same population 23 years after the first hybridization event occurred. We found 28 active colonies inhabiting 92 tree cavities surveyed in a 5.14 km^2^ area, resulting in a colony density of 5.4 colonies/km^2^. Of these 28 colonies, 25 were of *A. m. scutellata* maternal ancestry, and three were of western European maternal ancestry. No colonies of eastern European maternal ancestry were detected, although they were present in the earlier samples. Nuclear DNA revealed little change in the introgression of *A. m. scutellata*‐derived genes into the population compared to previous surveys. Our results suggest this feral population remains an admixed swarm with continued low levels of European ancestry and a greater presence of African‐derived mitochondrial genetic composition.

## Introduction

The Africanization of the honey bee (*Apis mellifera*) in North America represents a model of how an invasive population of a subspecies can interact with a resident population of a different subspecies to create a zone in which hybrid populations of a species complex can exist (Pinto et al. 2005). Africanized honey bees in the southern United States are derived from the sub‐Saharan subspecies *A. mellifera scutellata*, which was introduced into Brazil from South Africa in 1956 and crossbred with honey bees of European descent to create a hybrid that would better tolerate and thrive in a tropical environment (Kerr 1967). After the escape of several swarms in 1957 (Nogueira‐Neto 1964; Kerr 1967), hybrid swarms rapidly dispersed northward throughout the American tropics (Taylor 1988; Rubink et al. 1990; Winston 1992), reaching North America in <40 years (Sugden and Williams 1990). The success of Africanized honey bees in the Americas has been attributed to a combination of ecological and genetic factors that have conferred them higher fitness compared to the resident European honey bees (reviewed by Pinto et al. 2004; Schneider et al. 2004). Some of these life history characteristics include higher reproductive rates, shorter developmental period, higher drone production, higher absconding rates, higher defensiveness against colony intruders, lower honey‐storing needs, and lower selectivity when choosing nest sites, especially use of smaller cavities (Winston et al. 1983; Schmidt and Hurley 1995; Caron 2001; Schneider et al. 2004).

In temperate subtropical regions of South and North America, a genetically complex hybrid zone composed of honey bees of African and European descent has reshaped the dynamics of the populations that resided in these areas prior to the arrival of Africanized honey bees (Lobo et al. 1989; Sheppard et al. 1991; Diniz‐Filho and Malaspina 1995; Diniz et al. 2003; Quezada‐Euán et al. 2003). Founder populations of species invading new territory, or of subspecies invading the territory occupied by a different subspecies, are rarely successful and typically go extinct (Williamson 1996). In a few cases, founder populations of both types of invasions get well established and thrive in their new environment. Successful invasive populations frequently compete for resources, with resident populations of either different species that occupy similar niches or different subspecies within a species complex (Pimentel et al. 2000), by responding rapidly to strong local selection pressures (Lee 1999; Huey et al. 2000). In the case of invasions by subspecies within a species complex, the invasive populations can either outcompete the resident populations, pushing them toward extinction (Holway 1999; Wauters and Gurnell 1999), or can hybridize with them, forming a novel genetic subspecies entity (Huxel 1999). As a consequence, a new taxon evolves either as a “hybrid swarm,” in which the genes of the invasive and resident populations are mixed (Childs et al. 1996; Huxel 1999), or as a genetically assimilated population, in which the genes of the original resident population may disappear over time (Levin et al. 1996; Rhymer and Simberloff 1996; Perry et al. 2001). Biotic factors (e.g., pathogens and parasites) and abiotic factors (e.g., harsh environmental conditions) can expedite the rate of genetic assimilation of a resident population with genes from the invasive population (Calvo‐Ugarteburu and McQuaid 1998; D'Antonio 2000; Rushton et al. 2000).

In the United States, honey bees comprise a unique admixture of introduced subspecies. Before the arrival of Africanized honey bees in 1990 (Rubink et al. 1996), feral and managed colonies were composed of a panmictic mixture of subspecies derived from eastern (*A. m. carnica, A. m. caucasica* and *A. m. ligustica*) and western (*A. m. iberiensis* and *A. m. mellifera*) European lineages (Rubink et al. 1990; Schiff et al. 1994; Kraus and Page 1995; Schiff and Sheppard 1996; Loper et al. 1999; Harpur et al. 2012). Interestingly, there is evidence that *A. m. scutellata*‐derived genes were introduced into the United States earlier than 1990, but that introduction was largely unsuccessful and those genes remained a very minor component of the admixed population (Schiff et al. 1994; Whitfield et al. 2006; Pinto et al. 2007). All of this changed in the last 25 years, as hybridization of honey bees with lineages of *A. m. scutellata* descent became very successful in southern regions of the United States (Whitfield et al. 2006; Pinto et al. 2007; and see Chapman et al. 2015 for a summary).

Taylor and Spivak (1984) described the expansion of the descendants of *A. m. scutellata* from the point of introduction in Brazil northward. The authors predicted that in the United States, these descendants would invade regions of the southernmost states and form a hybrid zone northward in a pattern similar to what was predicted for the southern regions of South America (Sheppard et al. 1991; Diniz et al. 2003). Despite their ubiquitous success in the tropics, however, Africanized honey bees have not spread to some areas of South and North America that were originally predicted to be within their future range (e.g., Taylor and Spivak 1984; Southwick et al. 1990).

The hybridization process was well documented as the migration of Africanized honey bees reached Mexico in 1986, with one particular population of feral honey bees in the Yucatan Peninsula having been carefully monitored for over a decade. Several studies reported that before 1986, this population was primarily of southeastern European ancestry with a small proportion of northwestern European origin. By 1989 the population appeared to have only very low levels of maternal gene flow from Africanized populations. By 1998, however, substantial nuclear and mitochondrial gene flow had occurred, with a majority of the mitochondrial and nuclear genes being of African descent (Rinderer et al. 1991; Quezada and Hinsull 1995; Quezada‐Euan et al. 1996; Quezada‐Euán and Medina 1998; Quezada‐Euán 2000; Clarke et al. 2001, 2002). The admixture patterns in that population after 1998 have not been reported, and the long‐term outcome of these hybridization events is unknown.

A similar process of hybridization in the United States was examined in a temporal study by Pinto et al. (2004, 2005), who conducted an 11‐year survey of the mitochondrial and nuclear DNA composition of feral honey bee colonies in South Texas. Interestingly, only five years after detection of the first Africanized swarms in 1993, there was a substantial *A. m. scutellata* maternal component in the invaded resident population. The gene flow was not unidirectional, however, as nuclear microsatellite loci showed bidirectional gene flow between the resident and the invasive populations. This process created a new “Africanized hybrid swarm,” an admixture of *A. m. scutellata*‐derived and European‐derived genes (Pinto et al. 2004, 2005). More recent studies of this admixture process conducted in other locations have shown that Africanized honey bees in the United States still possess some European genetic ancestry, and yet over time, alleles from European haplotypes are being lost due to Africanization, with the greatest loss to alleles from the Eastern lineage (Schneider et al. 2004; Whitfield et al. 2006; Chapman et al. 2015). It is not known what happened to the admixed swarm of feral honey bees in South Texas since the last survey in 2001, however.

In the current study, conducted in the summer of 2013, we reevaluated the genetic structure of the same feral honey bee population in South Texas. We visited tree cavities previously identified as being used by feral honey bees (Pinto et al. 2004, 2005) and sampled workers from all active colonies identified within the sampling area to assess the current pattern of hybridization and genetic introgression. We then evaluated whether the gene pool in this population continues to be composed of recombinant haplotypes with a substantial European genetic contribution at the mitochondrial and nuclear level as found by Pinto et al. (2005), or if African‐derived genes have now fully replaced those of European‐derived ancestry. Our study was conducted after an extreme drought in 2011, which potentially influenced the distribution and genetic composition of the previously studied admixed population.

## Methods

### Study site

This study was conducted at the Welder Wildlife Refuge (WWR), San Patricio County, Texas (28°07′ 18″, −97°26′34″). Located between the South Texas Plains and the Gulf Prairies and Marshes ecoregions, the WWR lies in a transitional zone with vegetation cover comprised mostly of live oak mottes, scattered mesquite, chaparral brushland, and open grassland (Drawe et al. 1978; Blankenship 2000). The climate is mostly humid, with hot summers (particularly in years of intense drought such as that observed in 2011), and cool winters (Blankenship 2000; Baum 2003; Baum et al. 2004).

### Sample collection, cavity occupancy, and colony density

A thorough survey of a 6.25 km^2^ area at the WWR led to the discovery of 109 trees housing feral honey bee colonies between 1991 and 2001 (Baum et al. 2005). All trees were individually labeled with numbered metal tags and their GPS coordinates were logged. Most colonies (85%) inhabited cavities of live oak trees, while the remaining colonies occurred in elm or fallen live oak trees (Baum et al. 2005). In June 2013 we returned to the WWR and surveyed a 5.14 km^2^ area within the original study site used by Baum (2003), excluding the riparian part of the previously surveyed area (where tree mortality was high due to the 2011 drought), which prevented us from accurately locating all of the previously occupied tree cavities. We discovered 89 of the 109 cavities reported earlier (Pinto et al. 2004; Baum et al. 2005) by matching GPS coordinates and numbered tags with our records. We identified all cavities that were currently occupied by feral honey bees from the 89 previously surveyed trees, and identified any new tree cavities with active colonies within the survey area, which were labeled and logged on the GPS unit. We collected 2–5 workers from the hive entrance of all active colonies, stored them in dry ice and transported them to the laboratory where they were kept frozen at –80°C for subsequent DNA analysis. We estimated the density of feral colonies occupying known tree cavities per km^2^ by noting the number of active colonies found within the 5.14 km^2^ surveyed area and compared it to the densities reported from previous surveys (Baum et al. 2005). We calculated the mean colony density from 1991 to 2013 as the average number of colonies per km^2^ ± one standard deviation of the mean (SDM).

### Reference populations

Honeybee workers representing two populations, one European‐derived and another *A. m. scutellata*‐derived, were used as reference populations. The European‐derived population (*n *=* *50 individuals, one from each of 50 colonies) was sampled from feral swarms trapped in South Texas between 1988 and 1990 prior to Africanization (Rubink et al. 1996; Pinto et al. 2005). The *A. m. scutellata*‐derived population (*n *=* *43 individuals, one from each of 43 colonies) was sampled in 2002 from feral swarms collected in São Paulo, Brazil, in an area near the initial release site of *A. m. scutellata* (Pinto et al. 2005). The reference populations are referred to as “European” and “Brazilian” throughout this article.

### DNA extraction and mitochondrial DNA analysis

Total DNA was extracted from the thorax of a single worker per colony (randomly selected from those collected) sampled at the WWR in 2013 using a QIAamp DNA Mini Kit (Qiagen Inc., Valencia, CA) according to the manufacturer's protocol. The extracted DNA templates were stored at −20°C until analysis. We analyzed one to three regions of the mitochondrial genome through polymerase chain reaction (PCR) in a stepwise manner as described by (Pinto et al. 2003, 2004). Briefly, we first amplified a 485‐bp section of the cytochrome *b* gene using the primers 5′‐TATGTACTACCATGAGGACAAATATC‐3′ and 5′‐ATTACACCTCCTAATTTATTAGGAAT‐3′ (Crozier et al. 1991; Pinto et al. 2003). We then amplified a 738‐bp section of the large ribosomal subunit (ls rRNA) gene using the primers 5′‐TTTTGTACCTTTTGTATCAGGGTTG‐3′ and 5′‐CTATAGGGTCTTATCGTCCC‐3′ (Hall and Smith 1991). Finally, we amplified a 1028‐bp section of the cytochrome oxidase I (COI) gene using the primers 5′‐GATTACTTCCTCCCTCATTA‐3′ and 5′‐AATCTGGATAGTCTGAATAA‐3′ (Crozier and Crozier 1993; Nielsen et al. 2000).

We performed single PCR amplifications in 50‐*μ*L total volumes using the AmpliTaq kit from Life Technologies (Grand Island, NY). Reactions contained the following reagents: 1X PCR Buffer II, 0.5 mmol/L MgCl_2_, 0.2 mmol/L dNTPs, 0.5 *μ*mol/L each of forward and reverse primers (sequences and references above), 2 units AmpliTaq DNA Polymerase, and 9.6 *μ*L genomic DNA. For cytochrome *b* and COI amplification, the PCR temperature protocol was 94°C for 3 min followed by 31 cycles of 94°C for 15 sec, 50°C for 15 sec, and 68°C for 50 sec for all three pairs of primers. After the final cycle, an additional 10 min of extension at 72°C was performed. The same PCR protocol was used for ls rRNA, except we used 53°C for the annealing temperature.

Following PCR, DNA segments amplified with the cytochrome *b*, ls rRNA, or COI primers were digested with the restriction enzymes *Bgl*II, *Eco*RI, or *Hinf*I (New England Biolabs, Ipswich, MA), respectively, using the temperature and buffer conditions recommended by the manufacturer. A total of 5 *μ*L of restriction digest was then electrophoresed on a 1% agarose/TBE gel, stained with ethidium bromide, and visualized under UV light.

Because mitochondrial DNA is maternally inherited and does not recombine during sexual reproduction, we refer to colony haplotypes as being of *A. m. scutellata*, eastern European, or western European descent. Workers that were scored as carrying *A. m. scutellata* mitochondria following the *Bgl*II digestion of the cytochrome *b* PCR‐amplified fragment were not amplified further (Crozier et al. 1991; Pinto et al. 2003). Workers that exhibited a non‐ *A. m. scutellata* haplotype using the cytochrome *b*/*Bgl*II restriction assay were then PCR‐amplified for ls rRNA and digested with *Eco*RI, which discriminates colonies of eastern European maternal ancestry from those of western European maternal ancestry (Hall and Smith 1991). Workers that exhibited a non‐eastern European haplotype using the ls rRNA/*Eco*RI assay were PCR‐amplified for COI and digested with *Hinf*I to discriminate whether colonies were of western European maternal ancestry, or belonged to the *A. m. lamarckii* subspecies (Crozier and Crozier 1993; Nielsen et al. 2000; Pinto et al. 2004).

### Microsatellite analysis

Genetic variation among individual workers was scored at 12 highly polymorphic microsatellite loci (A7, A8, A14, A28, A35, A43, A79, A88, A107, A113, IM, and ED1) as described previously (Estoup et al. 1994, 1995; Oldroyd et al. 1995; Rowe et al. 1997; Garnery et al. 1998; Pinto et al. 2005). PCR reactions were performed following the protocol described by Boutin‐Ganache et al. (2001). Briefly, single PCR amplifications were performed in 10 *μ*L total volume containing 0.1 U *Taq* DNA polymerase (Life Technologies, Burlington, ON, Canada), 1.5 mmol/L MgCl_2_, 0.2 mmol/L of each dNTPs, 1.5 pM of each primer, and 125 ng of genomic DNA. Forward primers for each marker were extended using a 21‐bp tail sequence (5′‐GCCTCGTTTATCAGATGTGGA‐3′) and were labeled with one of three fluorescent dyes (HEX, 6‐FAM, or NED; Applied Biosystems, Foster City, CA). PCR cycling followed the protocol listed in Touch‐Down I by Renshaw et al. (2006). Briefly, initial denaturation occurred at 95°C for 3 min, with 12 cycles of denaturation at 95°C for 30 sec, annealing (beginning at 60°C less 0.5°C per cycle) for 1 min, and extension at 72°C for 4 min. Then, 30 cycles of denaturation were run at 95°C for 30 sec, 52°C for 1 min, extension at 72°C for 4 min, and final extension at 72°C for 10 min. The products were then separated on a 6% polyacrylamide gel under denaturing conditions (Boutin‐Ganache et al. 2001). Amplicons were visualized on an ABI 377 automated sequencer with a 400 HD Rox internal size standard (Applied Biosystems). The software Genotyper v.2.5 (Applied Biosystems) was used for allele identification and comparison.

To correct for the additional length of tail primers used in these studies, allele sizes were standardized to the allele size ranges of loci across the previous years (e.g., 1991–2001) by subtracting 21 bp from each allele. To test for consistency in allele sizes between individuals that were genotyped using the fluorescently labeled microsatellites and the tail‐labeled primers, a total of 10 bees were genotyped, six from one reference colony, and four from another reference colony. To ensure that alleles were scored the same way in this study and in Pinto et al. (2005), calibration was performed using full sisters taken from the colonies genotyped earlier. Every allele identified and assigned in 2013 was scored in agreement with Pinto et al. (2005).

### Statistical analysis

To determine the degree of genetic differentiation among populations at the WWR from 1991 to 2013, pairwise multilocus *F*_ST_ values were computed according to Weir and Cockerham (1984) using GENEPOP. Departures from zero were then tested by 1000 permutations using GENETIX version 4.04 (Belkhir et al. 2002). Differences in average unbiased gene diversity between pairs of samples were assessed by Wilcoxon's signed‐rank test (Snedecor and Cochran 1978). The individual‐based Bayesian clustering algorithm implemented in the software STRUCTURE 2.3.3 was employed to infer membership proportions (*Q*) in the WWR honey bee colonies and the reference populations of European and Brazilian descent (Pritchard et al. 2000). The number of ancestral clusters (*K*) was estimated using the admixture ancestry and correlated allele frequency models with the unsupervised option. STRUCTURE was set to 750,000 Markov chain Monte Carlo iterations after an initial burn‐in of 250,000. Twenty independent runs for each *K* (from 1 to 6) were performed to confirm consistency across runs. The output was exported into STRUCTURE HARVESTER web v0.6.94 (Earl and Von Holdt 2012). The Greedy algorithm, implemented in the software CLUMPP 1.1.2b (Jakobsson and Rosenberg 2007), was used to align the 20 runs for each *K*. The means of the permuted results were plotted using the software DISTRUCT 1.1 (Rosenberg 2004). The optimal *K* value was determined using Evanno's Δ*K* method in STRUCTURE HARVESTER web v0.6.94 (Evanno et al. 2005; Earl and Von Holdt 2012).

## Results

### Cavity occupancy and colony density

**Figure 1:**
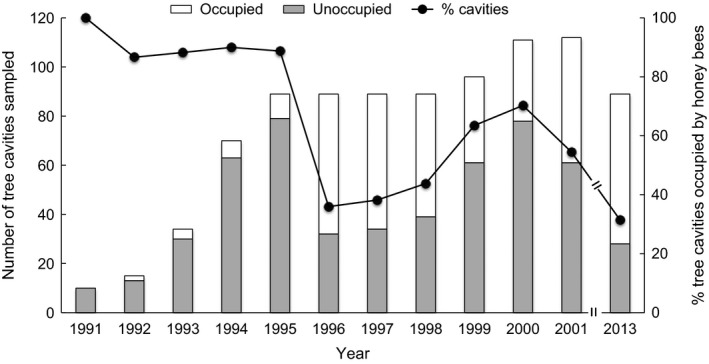
Distribution from 1991 to 2013 (represented by stacked bars) of discovered tree cavities that were either occupied (white bars) or unoccupied (gray bars) by feral honey bee colonies in a 6.25 km^2^ area surveyed 1991–2001 and a 5.14 km^2^ area surveyed in 2013 at the Welder Wildlife Refuge, San Patricio County, TX. The percentage of cavity occupancy by year is represented by the black dots on the secondary *y*‐axis, with a broken line between 2001 and 2013 to show that no data were collected during the 12‐year span. All values from 1991 to 2001 are reported here as the sum of all new and previously discovered trees inhabited by honey bee colonies as reported in Table 1 by Pinto et al. (2004). Due to sampling limitations, these surveys did not identify new tree cavities that could potentially foster colonies but were unoccupied at the time of sampling.

We surveyed 89 previously identified trees for the presence of feral honey bee colonies within a 5.14 km^2^ sample area in 2013 (see Table 1, [9]), or 78.8% of the total tree cavities that had been explored previously at the WWR (Pinto et al. 2004; Baum et al. 2005). We found three additional trees within the study area housing colonies that had not been identified in previous surveys. Thus in total, we surveyed 92 tree cavities in 2013, 28 of which were occupied by honey bees (Fig. 1). We did not find any new empty tree cavities that seemed to have occupied colonies in the past. The density of identified trees that were occupied by honey bees when surveys were performed ranged from 1.6 trees/km^2^ in 1991 to 17.3 trees/km^2^ between 1991 and 2013 (Table 1). The percentage of the total number of identified tree cavities that were occupied by honey bee colonies when surveys were conducted was 70.3% in 2000, 54.5% in 2001, and 34.5% in 2013. Colony density (only accounting for the trees surveyed each year) was 12.5 colonies/km^2^ in 2000, 9.8 colonies/km^2^ in 2001, and 5.4 colonies/km^2^ in 2013 (Table 1). The 12‐year average colony density from 1991 to 2013 was 7.1 ± 3.74 colonies/km^2^.

**Table 1:** Spatial and temporal patterns of tree cavity occupancy and colony density from 1991 to 2001 based on a 6.25 km^2^ study area and again in 2013 based on a 5.14 km^2^ study area at the Welder Wildlife Refuge, San Patricio County, TX

Year	No. of tree cavities surveyed^a^	No. of colonies inhabiting tree cavities	Colony density (no./km^2^)^b^
1991	10	10	1.6
1992	15	13	2.1
1993	34	30	4.8
1994	70	63	10.1
1995	89	79	12.6
1996	89	32	5.1
1997	89	34	5.4
1998	89	39	6.2
1999	96	61	9.8
2000	111	78	12.5
2001	112	61	9.8
2013	89	28	5.4
1991–2001 average colony density ± SDM			7.3 ± 3.88
1991–2013 average colony density ± SDM			7.1 ± 3.74

a Cavity occupancy data from 1991 to 2001 are reported here as the sum of all established and new colonies inhabiting tree cavities as reported in Table 1 by Pinto et al. (2004).

b Colony density data from 1991 to 2001 are reported similarly to Baum et al. (2005) except that their number of colonies/km^2^ included colonies in tree cavities plus colonies captured in swarm traps, while our data set only includes feral colonies occupying tree cavities as reported also by Pinto et al. (2004).

### Mitochondrial DNA diversity

**Figure 2:**
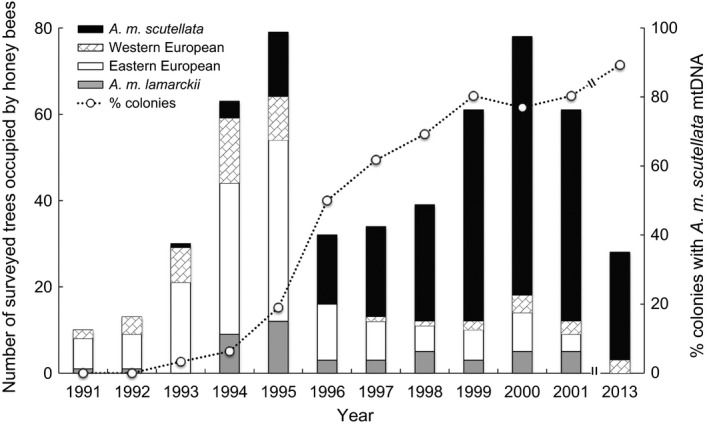
The maternal mitotypes of feral honey bee colonies at the Welder Wildlife Refuge, San Patricio County, TX, from 1991 to 2001, and again in 2013. Gray bars correspond to colonies with mitochondrial DNA belonging to *Apis mellifera lamarckii*, white bars correspond to cavities occupied by colonies of eastern European maternal descent, diagonal lines represent cavities occupied by colonies of western European maternal descent, and black bars represent the number of cavities occupied by colonies of *A. m. scutellata* descent. The percent of tree active cavities occupied by colonies of *A. m. scutellata* descent over time is represented by the dotted line with white circles (secondary *y*‐axis), with a broken line between 2001 and 2013 to show that no data were collected during that 12‐year span. Data from 1991 to 2001 are the sum of all established and newly found tree cavities occupied by colonies, as reported in Table 1 by Pinto et al. (2004).

Of the 28 colonies surveyed in 2013, workers from 25 colonies exhibited *A. m. scutellata*‐derived maternal ancestry, while workers from the other three colonies exhibited western European‐derived maternal ancestry. We did not find any colonies with either eastern European or *A. mellifera lamarckii* mitochondrial DNA (Table 2, Fig. 2). The proportion of feral honey bee colonies of *A. m. scutellata‐*derived haplotypes in 2013 was 0.89, which was significantly greater than the proportion of *A. m. scutellata*‐derived haplotypes found in the colonies surveyed from 1991 to 2001 (*P* ≤ 0.005, see Table 2 for Fisher's exact *P*‐values; Fig. 2).

**Table 2:** Summary of the number of honey bees with maternally derived ancestry from *Apis mellifera lamarckii*, eastern European, western European, and *A. m. scutellata* inhabiting tree cavities at the Welder Wildlife Refuge, San Patricio County, TX, from 1991 to 2013. Fisher's exact test was used to calculate the two‐tailed statistical difference between the proportion of colonies with *A. m. scutellata‐derived* maternal ancestry found in 2013 compared to the same proportion found in colonies surveyed from 1991 to 2001. See “Methods” for details

Year^a^	Number of honey bees of each maternally derived haplotype					Fisher's exact test *P*‐value
	*Apis mellifera lamarckii*	Eastern European	Western European	*Apis mellifera scutellata*	Total	
1991	1	7	2	0	10	<0.0001
1992	1	8	4	0	13	<0.0001
1993	0	21	8	1	30	<0.0001
1994	9	35	15	4	63	<0.0001
1995	12	42	10	15	79	<0.0001
1996	3	13	0	16	32	<0.0001
1997	3	9	1	21	34	<0.0001
1998	5	6	1	27	39	<0.0001
1999	3	7	2	49	61	0.005
2000	5	9	4	60	78	0.0001
2001	5	4	3	49	61	0.005
2013	0	0	3	25	28	

a Data from 1991 to 2001 are reported here as the sum of all established and new colonies found in tree cavities as reported in Table 1 by Pinto et al. (2004).

### Nuclear structure

**Figure 3:**
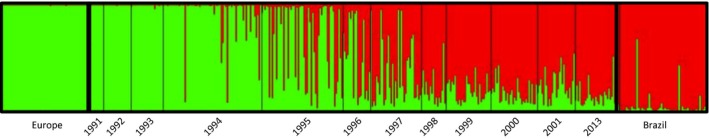
Assignment of individual honey bees inferred from 12 microsatellite loci using the software STRUCTURE. Each of the 456 individuals included in the analysis is represented by a vertical bar partitioned into two (*K* = 2) segments, represented by green and red, corresponding to membership proportions in each of the two optimal *K* clusters, as estimated by Evanno's Δ*K* (Evanno et al. 2005). Black lines separate honey bee samples from the Welder Wildlife Refuge, San Patricio County, TX, by year (from 1991 to 2001, and 2013) and the reference samples from Europe (green) and Brazil (red).

**Figure 4:**
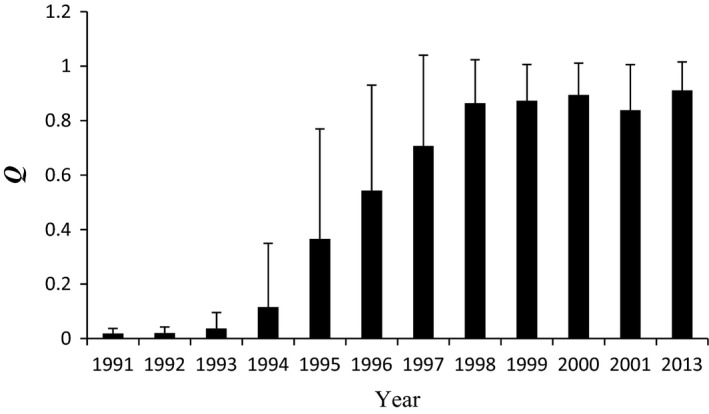
Average membership proportions (*Q*), and standard deviation, assigned in the Brazilian reference cluster by STRUCTURE for individual values at the optimal *K* = 2 (see Fig. 3) over time.

We did not detect null alleles, large allele dropout, or stuttering, for any microsatellite locus. Membership proportions inferred by STRUCTURE from the 12 microsatellite loci for the WWR population and reference populations representing the pre‐Africanization European‐derived (i.e., “European”) and *A. m. scutellata*‐derived (i.e., “Brazilian”) populations are shown in Fig. 3 for the optimal *K* = 2 estimated using Evanno's Δ*K* (Fig. S1). Introgression of the *A. m. scutellata*‐derived genes into the resident WWR population is evident from the increasing proportion assigned to the Brazilian reference cluster (red lines in Fig. 3) of each honey bee individual evaluated in successive sampling years (Fig. 3). From 1991 to 1993, most individuals (75%) were assigned with high posterior probability (≥0.98) to the European reference cluster (green lines in Fig. 3). From 1993 on, when the first colony with *A. m. scutellata*‐derived maternal descent was detected, the membership proportion (*Q*) in the Brazilian reference cluster increased dramatically to above 0.8, with the average *Q* values approaching 0.91 in 2013 (Fig. 4).

## Discussion

The feral honey bee population at the WWR in South Texas represents a biological model of survival under adversity. The population was invaded from the south by Africanized honey bees in the early 1990s. It was also negatively impacted from the east with the arrival of the ectoparasitic mite *Varroa destructor* in the 1990s (Kraus and Page 1995) and the gut pathogen *Nosema ceranae* in the early 2000s (Wenner and Bushing 1996; Chen et al. 2008). The feral honey bee population at the WWR has gone through a period of intense genetic change over the last two decades. By 2013 its mitochondrial lineage had become 89% Africanized, retaining only a small proportion of European haplotypes, and 91% of its nuclear genome aligned with that of the Brazilian reference population instead of the European‐derived nuclear genome they exhibited in 1991.

From 1991 to 2001, the average honey bee colony density at the WWR was 7.3 ± 3.9 colonies/km^2^ (range = 1.6 to 12.6 colonies/km^2^). The density observed in 2013 of 5.4 colonies/km^2^ falls in the middle of this range, within one standard deviation of the average (Table 1). It is remarkable that despite strong environmental stressors, including the historically devastating regional drought in 2011 (Combs 2012), colony density in 2013 remained within the range reported earlier at the WWR (Baum 2003; Baum et al. 2005). However, the real colony density in our sampling area could be slightly different from our results either because we did not record all new unoccupied cavities (which would decrease the density of occupied tree cavities per unit area), or because we did not locate all newly occupied cavities (which would increase the number of active colonies per unit area).

Despite the potential sampling error in calculating true colony density, our estimate for 2013 is similar to other studies in areas with suitable and unsuitable patches of colony nesting habitat, including surveys in Panama (4–7 colonies/km^2^, Boreham and Roubik 1987), New York (2.7 colonies/km^2^, Morse et al. 1990), Mexico (6 colonies/km^2^, Ratnieks et al. 1991), and Botswana (4.2 colonies/km^2^, McNally and Schneider 1996). Our observed colony density in 2013 was roughly one order of magnitude higher than the density reported by Arundel et al. (2014) in Victoria, Australia (0.1–0.5 colonies/km^2^), and was nearly two times higher than the highest density (3 colonies/km^2^) that Hinson et al. (2015) reported for feral honey bees in undisturbed habitats in Southeastern Australia, however. Results from all these surveys confirm that different landscapes will accommodate different maximum colony densities depending on the biogeophysical properties of each habitat (Hinson et al. 2015).

The 12 microsatellite loci that were used for the reported genomic DNA analysis serve as a proxy for the evaluation of neutral variation in the nuclear genome. Bayesian clustering analysis showed that the ancestry of the WWR feral honey bee population changed from being almost completely European in the early 1990s to being almost completely African over the course of the next 12 years. While the high proportion of mtDNA of African ancestry shows the successful introgression of genes of African descent into this population, by 2013 the WWR population was not yet of pure African descent. Genetic assignment of individuals by the program structure showed only one individual having a hybrid nuclear genome with roughly half of its genes descending from Europe, and another three individuals having nuclear genomes with roughly one‐fourth of their genome from a European descent (Fig. 3). A small proportion of European‐derived genotypes were retained in the remaining 24 individuals. These results suggest that the new combinations of genes produced by recombination are likely adaptive for the biogeophysical conditions of the WWR, as these combinations clearly remained successful in the decade since the population was last examined. The four colonies with noticeable European‐derived genomes could be the result of matings between hybrid queens with drones of European descent coming from nearby managed colonies, as has been reported in other regions with strong but still incomplete Africanization of honey bee populations (Branchiccela et al. 2014). Given that there are no current records of any beekeeping operations near the WWR, this idea remains to be tested.

The presence of colonies with different assigned proportions of African‐ and European‐derived genome found by the structure analysis is consistent with the report of Pinto et al. (2005). Prior to 1992, the nuclear makeup of the WWR population was not significantly different from honey bees of European‐only ancestry (*F*_ST_ = 0.108, *P *=* *0.131; Table S1). By 1992, when Africanized swarms began to colonize South Texas, the WWR honey bee population became a unique mixture significantly different from both the European and the Brazilian reference populations, as measured by the *F*_ST_ values (Table S1). In 2013, the population remained significantly different from the reference European population (*F*_ST_ = 0.096; *P *=* *0.001; Table S1). Interestingly, whereas the maternal lineage of the WWR population was almost fully Africanized by 2013, the nuclear *F*_ST_ values remained relatively unchanged from 1997 (*F*_ST_ = 0.014; *P *=* *0.001; Table S1) to 2013 (*F*_ST_ = 0.015; *P = *0.001; Table S1).

The high proportion of colonies exhibiting mtDNA of *A. m. scutellata* ancestry in 2013 showed that the African maternal lineage in the WWR honey bee population is more common than that of any European subspecies. However, because we only sampled 28 colonies, and the frequency of colonies with maternal ancestry from European subspecies was already low in previous surveys that sampled 3–4 times more colonies than us, there could be more colonies with European maternal lineage in the sampling area that we were unable to find in 2013. Nevertheless, the continued prevalence of *A. m. scutellata* maternal lineages in the surveyed population may have outcompeted the eastern European and the *A. m. lamarckii* maternal lineages, which had been observed until 2001, albeit in small numbers. Complete loss of haplotypes belonging to eastern European and *A. m. lamarckii* subspecies has been widely observed, particularly in South America, where the only European haplotype that has remained after Africanization is the dark European honey bee, *A. m. mellifera* (see fig. 2C in Whitfield et al. 2006; Whitfield 2007). The overall decrease in non‐*A. m. scutellata* maternal lineages is not surprising, given that Africanized honey bees are extremely successful at expanding their territory and outcompeting other subspecies for floral resources and nesting sites if that territory falls within their ecological boundary (Michener 1975; Caron 2001; Caron and Connor 2013).

The process of genetic recombination of the WWR honey bee population raises some interesting questions: Are some of the new allelic combinations found in the “African hybrid swarm” adaptive? If so, do they confer fitness advantages to the hybrid individuals compared to the parental haplotypes? What characteristics does the “new” honey bee possess in terms of fecundity, fertility, disease resistance, longevity, or productivity? Interestingly, a recent study of the same honey bee population at the WWR showed that the levels of the microsporidian gut pathogens *Nosema apis* and *N. ceranae* have remained relatively low over the last 20 years compared to reported *Nosema* spp. levels in managed colonies (Rangel et al. 2015). This suggests that feral Africanized honey bees might be more tolerant to pathogens, perhaps because they are better adapted genetically to combat infections, but this idea remains to be explored. More studies are needed looking at the adaptive fitness of feral honey bees in natural settings.

In conclusion, our study found that the feral honey bee population at the WWR remains a hybrid swarm, with the majority of its mitochondrial genes being of African descent. Their genomic DNA continues to be a stable mixture of European‐ and African‐descendent genes, representing still one of the most striking biological invasions documented to date in the United States. This mixture perhaps has contributed to the success of this population in overcoming strong environmental pressures.

## Conflict of Interest

The authors declare that they have no conflict of interest.

## Supporting information


**Figure S1.** Graphical display of Evanno's Δ*K* (Evanno et al. 2005) that is used to infer the optimal *K* for the analysis of the Welder Wildlife Refuge, San Patricio County, TX, and reference populations (of European or Brazilian descent) of honey bees in 2013 (see Fig. 3) using 12 microsatellite loci.

**Table S1**. Proportion of multilocus estimates of *F*_ST_ (above diagonal) and *P*‐values of genotypic differentiation (below diagonal) for feral honey bee colonies collected at the Welder Wildlife Refuge (WWR), San Patricio County, TX, between 1991 and 2001 and again in 2013.
