# Supplementary material for: Africanization of a feral honey bee (Apis mellifera) population in South Texas: does a decade make a difference?
Source: Ecol Evol. 2016 Mar 2;6(7):2158–69. doi: 10.1002/ece3.1974 (PMC4782243; doi:10.1002/ece3.1974)
Supplement: Supplementary file 1 — Figure S1. Graphical display of Evanno's ΔK (Evanno et al. 2005) that is used to infer the optimal K for the analysis of the Welder Wildlife Refuge, San Patricio County, TX, and reference populations (of European or Brazilian descent) of honey bees in 2013 (see Fig. 3) using 12 microsatellite loci. [file ECE3-6-2158-s001.docx]

**
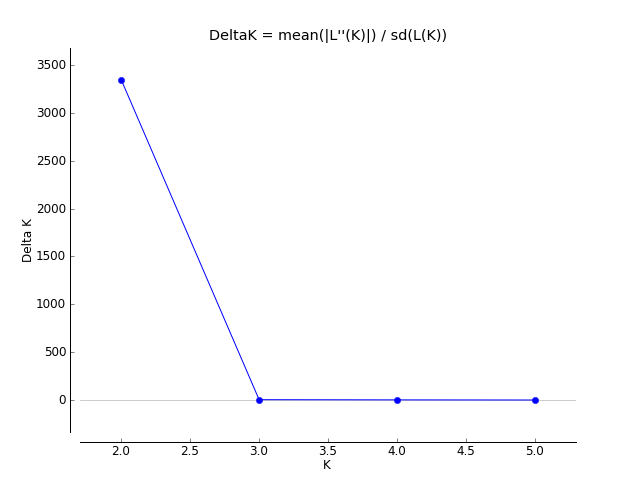
**

**Figure S1.** Graphical display of Evanno’s ΔK (Evanno *et al.* 2005) that is used to infer the optimal K for the analysis of the Welder Wildlife Refuge, San Patricio County, TX and reference populations (of European or Brazilian descent) of honey bees in 2013 (see Figure 3) using 12 microsatellite loci.
